# Promoting a global culture of respectful maternity care

**DOI:** 10.1186/s12884-023-06118-y

**Published:** 2023-11-17

**Authors:** Shuby Puthussery, Wubet Alebachew Bayih, Hilary Brown, Raymond Akawire Aborigo

**Affiliations:** 1 Maternal and Child Health Research Centre, Institute for Health Research, University of Bedfordshire, Park Square Rm B201, Luton, Bedfordshire LU1 3JU UK; 2 Department of Maternal and Neonatal Health Nursing, Debre Tabor University, Debre Tabor, Ethiopia; 3 Department of Epidemiology and Preventive Medicine, Monash University, Melbourne, VIC Australia; 4 Department of Health and Society, University of Toronto Scarborough, Toronto, Canada; 5 Dalla Lana School of Public Health, University of Toronto, Toronto, Canada; 6 Navrongo Health Research Centre, Navrongo, Ghana

## Abstract

Respectful maternity care (RMC) - a fundamental human right for all women - prioritizes autonomy and rights of pregnant and birthing women throughout the entire childbirth journey. Despite increasing acknowledgment of the importance of RMC for optimal maternal and new-born outcomes, women often experience disrespectful and abusive practices during pregnancy and childbirth. This Editorial points to the need for development of international guidelines for the implementation of RMC programs globally.

## Main text

Respectful maternity care (RMC) is a fundamental human right for all women. RMC is defined as care that maintains dignity, privacy, and confidentiality of pregnant and birthing women, ensures freedom from harm and mistreatment, and enables informed choice and continuous support during labor and birth [1, 2]. RMC prioritizes autonomy and rights of women throughout the entire childbirth journey and calls for the organization and management of health systems in a manner that places respect for women’s human rights at the forefront of their care [2].

RMC should be a primary goal for all healthcare providers, but women often experience disrespectful and abusive practices during pregnancy and childbirth across global contexts, especially in low and middle-income countries [3–6]. While some women tend to view such practices as ‘normative’, acknowledging that healthcare professionals did not intend to cause harm by such behaviors, others describe these practices as devaluing and dehumanizing to their sense of dignity, expressing an inherent lack of choice and an underlying sense of helplessness [4].

Studies have demonstrated links between disrespectful and abusive care and adverse maternal and infant health outcomes such as postpartum haemorrhage, physical injuries to the mother or new-born, and psychological consequences such as post-traumatic stress, suicidal ideation, and feelings of helplessness [4, 7–9]. It has been indicated that such practices can hamper mothers’ trust in the health system, leading to poor health-seeking behavior, delayed access to health facilities and an increase in the rate of births with unskilled attendants [5, 10].

Accumulated evidence has supported the development of international guidelines for the implementation of respectful maternity care programs [11, 12]. These guidelines include evidence-based recommendations that aim to improve the quality of intrapartum care [2] and postnatal care [13] with the ultimate aim of improving maternal and new-born health and well-being. All the guidelines emphasize giving women a positive maternity care experience as a key endpoint for women who access the service. This experience is defined as one in which everybody, including significant others who accompany the woman to the health facility, receive consistent support from motivated health workers who respect the cultural context in which they operate.

Despite increasing acknowledgment of the importance of RMC for optimal maternal and new-born outcomes, there is limited evidence on the uptake of RMC, particularly in low-resource settings. This knowledge gap is driven by the failure of routine health information systems and facility assessments to capture data on RMC practices [14]. However, despite this limitation, existing studies, which frequently use in-person observations (e.g., of labor and delivery settings), show sub-optimal application of RMC. For example, observations have shown that while women tended to be treated with dignity overall, numerous poor interactions were identified, from a lack of provision of adequate information to birthing women to physical and verbal abuse [14].

Thus, several challenges to the application of RMC remain. One primary challenge is a lack of evidence-based health care provider training and resources on RMC, which leads to poor awareness and knowledge of RMC standards and practices as well as negative attitudes and poor adherence [15]. There is also a lack of education for women themselves, in terms of their awareness of their right to information, informed consent, and respect for their preferences and choices [14]. In low-resource settings in particular, additional challenges are frequently infrastructural and include inadequate facilities for implementation of RMC practices, such as insufficient space (e.g., to provide birthing women with privacy or free movement while they labor) and inadequate staffing (e.g., to support patient preferences and shared decision-making, and to communicate patient needs across providers) [15].

It is vital to prevent disrespect and abuse in maternity care to ensure the well-being of pregnant and birthing women and their babies. Respecting women’s right to freedom from harm, providing compassionate care, and fostering a supportive environment are essential for positive birth experiences and healthy outcomes [1, 2]. Therefore, this Collection, ‘Respectful maternity care,’ aims to provide the global scientific community with more granular evidence about the state of respect, autonomy, dignity, privacy and confidentiality in the continuum of maternity care. Moreover, evidence on feto-maternal and neonatal health impacts of maternal disrespect and abuse are welcome in the Collection. The role of RMC in mitigating global maternal and neonatal mortality and morbidity, and its relationship with the Sustainable Development Goals agenda, is a key part of the Collection’s purpose. Notably, barriers to the implementation of respectful maternity care and strategic approaches to deal with the challenges will be covered. Thus, it is our earnest call to researchers whose work aligns with the purposes and aims of the Collection, to advance the scientific discourse with their evidence for improving maternal and neonatal health globally.

## Data Availability

Not applicable.

## Abbreviations

RMC: Respectful maternity care
WHO: World Health Organisation
